# Partners in time: Cospeciation and host-switching shape the evolution of *Leishmania* parasites

**DOI:** 10.1371/journal.pntd.0013814

**Published:** 2025-12-23

**Authors:** Alicia Rojas, Jairo Alfonso Mendoza-Roldan, Paula Alfaro-Segura, Alberto Solano-Barquero, Domenico Otranto

**Affiliations:** 1 Centro de Investigación en Enfermedades Tropicales, University of Costa Rica, San José, Costa Rica; 2 Laboratory of Helminthology, Faculty of Microbiology, University of Costa Rica, San José, Costa Rica; 3 Department of Veterinary Medicine, University of Bari, Bari, Italy; 4 Department of Veterinary Clinical Sciences, City University of Hong Kong, Hong Kong, China; Advanced Centre for Chronic and Rare Diseases, INDIA

## Abstract

**Background:**

*Leishmania* is a neglected tropical parasite that may affect millions of people worldwide, causing infections that range from subclinical to life-threatening in many animal species, including humans. The four subgenera described (i.e., *Leishmania*, *Mundinia*, *Sauroleishmania* and *Viannia*) exhibit distinct biological and ecological characteristics. The genus likely originated during the Mesozoic era, with hypotheses suggesting a Palearctic, Neotropical, or Supercontinent origin. Understanding the evolutionary history of *Leishmania* parasites may clarify host specificity, geographic distribution, and vector associations across subgenera.

**Methodology and key findings:**

In this study, we analyzed *Leishmania* evolutionary associations with vertebrate hosts as well as sand fly vectors using a combination of cophylogenetic and maximum parsimony approaches. A significant phylogenetic congruence was found between *Leishmania* and its vertebrate hosts using PACo (*p = *0.0001, m^2^_XY_=0.5419) and ParaFit (*p = *0.0034, ParaFit Global statistic = 0.012), with several host-switching and duplication events (*p = *0.0298), particularly involving *Leishmania*-humans associations. A cospeciation event was also predicted at a higher taxonomic level, corresponding to the divergence of mammals from reptiles and the subsequent separation of the *Leishmania, Viannia* and *Mundinia* subgenera from *Sauroleishmania.* Similarly, significant cophylogenetic signals were observed between *Leishmania* and sand fly hosts using PACo (m^2^_XY_=1.3988, *p* = 0.0001) and ParaFit (ParaFit Global = 0.062, *p = *0.0001) functions. Cospeciation events were again predicted at higher taxonomic levels in the divergence of New World and Old World sand flies, with *Viannia* species and *Leishmania, Sauroleishmania* and *Mundinia* species associated with the latter (*p* = 0.01).

**Conclusions:**

Our findings support the Supercontinent hypothesis and emphasize the relevance of the historical biogeography in *Leishmania* diversification. This comprehensive cophylogenetic analysis enhances our understanding of *Leishmania* origins and diversification, offering insights into host specificity, vector adaptation, and the long-term maintenance of its digenetic life cycle.

## Author summary

This study highlights cospeciation as central for *Leishmania* parasites and sand fly coevolution, while host-switching and duplication are more common with vertebrates. Strong phylogenetic congruence was observed between the *Viannia* subgenus and humans, the *Sauroleishmania* subgenus and lizards, and the *Leishmania* subgenus and rodents, supporting both recent and ancient co-divergence. Our findings support the Supercontinent hypothesis and emphasize the role of historical biogeography in *Leishmania* diversification. *Mundinia* species demonstrated adaptability to both sand flies and biting midges, suggesting a broader vector range. Overall, this analysis clarifies *Leishmania* origins, host specificity, and vector adaptation, leading to the development of targeted interventions to reduce infections in vulnerable groups

## Introduction

*Leishmania* is a genus of vector-borne parasites (family Trypanosomatidae) comprising *Leishmania, Sauroleishmania, Mundinia* and *Viannia* subgenera. Of the 53 *Leishmania* spp. described [1], approximately 20 are considered pathogenic to humans [2], causing neglected tropical diseases in 98 countries. About 350 million people are at risk of infection with 1.3 million cases occurring every year [2] in tropical and subtropical regions [3]. Moreover, this parasite can infect a myriad of mammal hosts with dogs, cats, rodents and other wild animal species acting as main reservoirs of the most pathogenic *Leishmania* spp. to humans [4].

Leishmaniasis is a zoonotic infection that occurs when, during a bloodmeal of a dipteran sand fly, parasites’ promastigotes are injected into host tissues. These then develop into their non-flagellated stage in the reticuloendothelial cells [4]. In particular, while *Leishmania, Mundinia* and *Viannia* subgenera have been associated with mammal hosts, *Sauroleishmania* is mostly linked to herpetofauna. These parasites have different niches inside their vertebrate hosts, leading to asymptomatic infections, cutaneous manifestations and even life-threatening conditions [5,6]. Accordingly, five main clinical presentations are recognized in humans: i) visceral leishmaniasis or kala-azar (VL) mostly caused by *Leishmania* (*Leishmania) infantum*; ii) post kala-azar dermal leishmaniasis (PKDL) produced mainly by *Leishmania (Leishmania) donovani,* but also by *L. infantum* [7]; iii) cutaneous leishmaniasis (CL) associated with several *Leishmania* spp. of the *Viannia, Mundinia* or *Leishmania* subgenus, including *L. tropica* [8]; iv) diffuse cutaneous leishmaniasis (DCL) produced by different species of the *Leishmania* subgenus, including *Leishmania mexicana* or *Leishmania aethiopica* [9]; and v) mucocutaneous leishmaniasis (MCL) induced by several species of the *Viannia* subgenus [8]. On the other hand, little is known about the clinical consequences of *Sauroleishmania* species in their reptilian hosts. Experimental infections with *Leishmania* (*Sauroleishmania*) *adleri* and *Leishmania* (*Sauroleishmania*) *tarentolae* have been conducted in various reptile species with no pathological effects observed [10], suggesting a long-term adaptation with these hosts.

Biogeographical segregation is apparent in *Leishmania* spp. distribution, since the *Viannia* subgenus is present predominantly in the New World and infects the hindgut of New World sand flies [4,11], whereas species of the *Sauroleishmania* subgenus have been restricted to the Old World [12], with few reports in South America [13]. Parasites of the *Sauroleishmania* subgenus use Old World sand flies such as *Sergentomyia* and *Phlebotomus* as invertebrate hosts [14]. On the other hand, the *Mundinia* subgenus is less understood, and has been reported in Australia, Ghana, Thailand and Martinique [15] where it is vectored by sand flies and biting midges. Finally, species of the *Leishmania* subgenus infect mostly the midgut of Old World sand flies but have also adapted to New World sand fly species [16]. The *Leishmania* subgenus mostly circulates in the Old World, except for *L. mexicana, Leishmania amazonensis* and *L. infantum* [12]. Interestingly, two variants of *L. infantum* have been described: one from the Old World and another from the New World [17]. The latter was likely introduced to the Americas 500 years ago, during the European colonization [18,19]. Therefore, an important question in *Leishmania* biology concerns its origin and evolution, which in turn, could provide insights into its association with specific vertebrate and invertebrate hosts, its geographic distribution, as well as predicted expansion in the Anthropocene, which is characterized by a changing environment.

According to the results of isoenzyme analyses, the origin of the *Leishmania* genus occurred during the Mesozoic era (i.e., before the separation of Gondwana) [20], at the same time that the divergence in sand flies of the New World and Old World sand flies occurred (i.e., 180–200 Million years ago (Mya)) [12]. More recent research using 200,000 informative sites from the parasite’s genomes, estimated the divergence of this genus from other trypanosomatids, such as *Trypanosoma cruzi* and *Trypanosoma brucei*, to have occurred 90–100 Mya [21]. Therefore, several hypotheses have been raised regarding the origin of *Leishmania.* Firstly, the “Palearctic hypothesis” suggests that *Leishmania* originated from Cretaceous lizards found in the Palearctic region, with *Sauroleishmania* proposed as the ancestral lineage to all other species [22]. In this scenario, Cretaceous lizards migrated to the Nearctic and Neotropics through land bridges and is supported by biogeographic and fossil data. The “Neotropical hypothesis” is based on sequence-based phylogenies and indicates that a predecessor of *L. donovani* and *Leishmania major* evolved from monoxenous parasites of insects in South America (46–36 Mya). In this hypothesis, the *Viannia* subgenus is ancestral to all species since the *Leishmania* subgenus may have originated in the Neotropics and spread approximately 24–14 Mya through the Bering Land Bridge [23]. According to the latter hypothesis, *Sauroleishmania* would have originated from mammal-associated *Leishmania* spp. Finally, the “Supercontinent or Multiple Origins hypothesis” indicates that *Leishmania* genus originated in Gondwana and later split into *Viannia* to the Neotropics and *Leishmania* and *Sauroleishmania* in Africa, approximately 90–100 Mya [24]. This is supported by multi-locus phylogenetic reconstructions [21]. Given that multiple lines of evidence have produced conflicting hypotheses about the origin of *Leishmania*, it is pertinent to examine the relationships between this parasite and its hosts from a coevolutionary perspective.

This study explores the evolutionary relationships between *Leishmania* and its vertebrate and invertebrate hosts by using three different strategies: i) cophylogenetic analysis based on patristic distances from host and parasite phylogenies, ii) cophylogenetic analysis using phenetic distances and iii) estimation of the coevolutionary events occurring between *Leishmania* and its vertebrate and invertebrate hosts. Our aim is to evaluate whether these biological and ecological relationships are supported by statistical testing of global fit and event-based methods. Understanding the evolutionary history of *Leishmania* is crucial for informing public health strategies aimed at controlling its spread.

## Materials and methods

### Sequence availability analysis

To determine which DNA markers are most widely represented among *Leishmania* species in GenBank, sequences originating from various cellular sources were systematically retrieved from database records. This approach facilitated the selection of markers for subsequent phylogenetic analysis. *Leishmania* sequences of nuclear, mitochondrial, ribosomal and kinetoplast origin were mined from GenBank under the following search criteria: RNA polymerase subunit II (POLR2), internal transcribed spacer 1 (ITS1), internal transcribed spacer 2 (ITS2), small subunit ribosomal, 18S, 5.8S, minicircle kinetoplast, cytochrome b, cytochrome oxidase subunit 1 (*cox*1) and heat shock protein 70 (hsp70), all listed in S1 Fig. These sequences were searched for parasites of the subgenus *Leishmania* (i.e., *Leishmania aethiopica, Leishmania amazonensis, Leishmania arabica, Leishmania donovani, Leishmania ellisi, Leishmania garnhami, Leishmania infantum, Leishmania major, Leishmania mexicana, Leishmania tropica* and *Leishmania turanica*), *Mundinia* (i.e., *Leishmania chancei, Leishmania enriettii, Leishmania macropodum, Leishmania martiniquensis, Leishmania orientalis* and *Leishmania procaviensis*), *Sauroleishmania* (i.e., *Leishmania adleri, Leishmania gymnodactyli, Leishmania hoogstraali* and *Leishmania tarentolae*) and *Viannia* (i.e., *Leishmania braziliensis, Leishmania guyanensis, Leishmania lainsoni, Leishmania naiffi, Leishmania panamensis,* and *Leishmania peruviana*). In addition, the number of genomes available for each of the species above mentioned was also surveyed in GenBank database. The number of sequences and genomes for each *Leishmania* spp. were recorded and visualized in PowerBI (Microsoft, USA). All sequences analyzed in this study were exclusively obtained from the GenBank database. No new sequence data were generated as part of this research. Additionally, information pertaining human or animal patients was not included, as the primary objective was to investigate the phylogenetic relationships between *Leishmania* parasites and their respective hosts.

### Phylogenetic analysis

#### Phylogeny with 18S, kDNA and POLR2.

**Fig 1:**
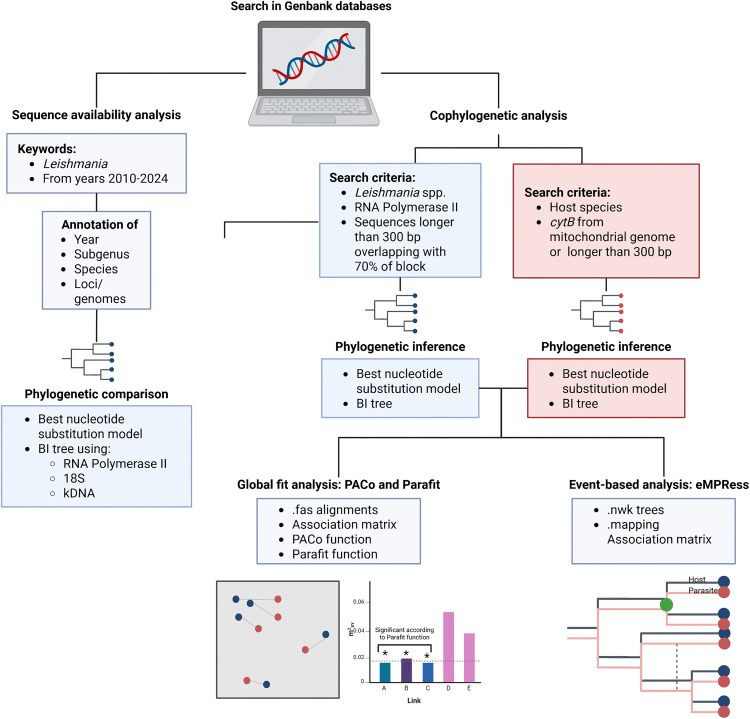
Pipeline of the analysis conducted. Created in BioRender. Mora, **J.** (2026) https://BioRender.com/is02atg.

By running the sequence availability survey, it was found that POLR2, ITS1 and 18S were the most represented molecular markers among *Leishmania* spp., as well as sequences most frequently aligned as a block. Therefore, Bayesian Inference phylogenetic trees were built with all those *Leishmania* sequences larger than 300 bp derived from different vertebrate hosts and that aligned as a block with other sequences as summarized in Fig 1. Sequences included in the analysis took into account all those *Leishmania* spp. for which a specific host was reported, fragments were larger than 300 bp and aligned to the fragment of majority of sequences. Moreover, sequences of the same *Leishmania* spp. were added if they met the above criteria and several different hosts were reported in GenBank.

Two datasets were analyzed: i) *Leishmania* sequences isolated from vertebrate hosts as verified from metadata in GenBank and ii) *Leishmania* sequences associated with sand fly hosts, which have been reported in the literature as competent vectors. This search strategy was applied to sand fly hosts since very few *Leishmania* spp. have specific sand fly hosts annotated in their GenBank metadata. In addition, *Leishmania* vertebrate and invertebrate hosts cytochrome b sequences larger than 300 bp were retrieved from GenBank, for constructing the host’s phylogenetic trees.

Sequences were aligned in MEGA7 [25] using the CLUSTALW algorithm [26] and the best nucleotide substitution model was estimated in JModelTest2 [27] using 11 substitution schemes, four processors, including invariant sites and gamma distribution, Maximum Likelihood optimized and Nearest Neighbour Interchange base tree search. *Endotrypanum monteroge*i*i* (AF009158) was used as an outgroup for the *Leishmania* spp. POLR2 tree, *Leptomonas costoris (*JQ359728) for the 18S, *Trypanosoma cruzi* (AF401100) for the kDNA, *Petromyzon marinus* (NC_001626) for the vertebrate host tree and *Culex quinquefasciatus* (MN540366) for the sand fly invertebrate host trees. The Jukes-Cantor with gamma distribution was chosen for the 18S dataset, the Tamura-Nei with gamma distribution for the POLR2 alignment and the Tamura 3-parameter with gamma distribution for the kDNA. In addition, the Time-reversible with gamma distribution was selected for the sand fly phylogenetic tree, whereas the General Time Reversible with gamma distribution was chosen for the vertebrate host tree. Bayesian Inference phylogenetic trees of *Leishmania* spp. and their associated hosts were built using the BEAST2.5 package [28]. Briefly, alignments were uploaded in BEAUTi v2.6.7 and 10^8^ Monte Carlo Markov Chains (MCMC) were set with sampling every 10^3^ trees and 25% of the trees were discarded as burning. Chain convergence was verified with effective sample sizes of each parameter larger than 300 in Tracer v1.7.2. Finally, trees were compiled in TreeAnnotator v2.6.4 and visualized in FigTree v1.4.4.

### Cophylogenetic analysis

Two distinct strategies were applied to assess the congruence between *Leishmania* parasites and their vertebrate and sand fly hosts using global fit methods (Fig 1), namely the Procrustes Approach to Cophylogeny (PACo) [29] and ParaFit [30]. These methods typically detect cophylogenetic signals, indicating that closely related parasites tend to associate with closely related hosts, though not necessarily in strict phylogenetic congruence. Additionally, we used the event-based method eMPRess [31] to infer the most likely coevolutionary events shaping the phylogenetic relationships between *Leishmania* species and their hosts. A limitation of the study was the use of different markers for building host and parasite phylogenies, which could affect divergence estimates. However, similar strategies have been used in other studies yielding reliable results [32,33]. In addition, only those *Leishmania-*host links with available POLR2 and complete information in GenBank were tested. For sand fly-associated *Leishmania* species, we also used Jane [34], as this algorithm accommodates parasites with multiple hosts, unlike eMPRess.

Each of these analyses was performed on three different relationships between *Leishmania* spp. and the following host categories, based on the available number of host-parasite associations: i) *Leishmania* and confirmed vertebrate hosts according to GenBank metadata, ii) *Leishmania* and confirmed vertebrate hosts as observed in GenBank metadata of each parasite sequence, with the addition of *L. gymnodactyli* and the Caspian bent-toed gecko *Tenuidactylus caspius* (syn. *Cyrtopodion caspium*); this association may be possible due to the phylogenetic closeness of this reptile with the confirmed hosts *T. caspius* (syn. *Gymnodactylus caspius*) and *Paralaudakia caucasia* (syn. *Agama caucasica*)*,* which did not have available *cyt*B sequences deposited in GenBank. This analysis was done to increase the number of *Sauroleishmania* spp. in the study, and iii) *Leishmania* sequences and sand fly species which have been reported as suitable hosts in the literature (S1 Table).

For PACo implementation, each *Leishmania* taxon and its corresponding host were assigned a unique identifier available in S1 and S2 Tables. The input included two phylogenetic trees (hosts and parasites in newick format) and a binary matrix encoding host-parasite associations (1 = associated, 0 = not associated). PACo calculated patristic distance matrices, which were then transformed into principal coordinate (PCo) matrices. The parasite PCo coordinates were superimposed onto the host PCo coordinates, which effectively controls for host phylogeny. Phylogenetic congruence was assessed using the Procrustes global fit statistic (m^2^XY) in asymmetrical mode (sym = FALSE). Significance was evaluated using 1,000 random permutations of the association matrix. In asymmetrical mode, significant host constraint on parasite phylogeny is inferred if fewer than 5% of the randomized m_2_XY values are lower than the observed value. The contribution of each host-parasite link to the global fit was determined via jackknife resampling, taking one link away from each randomization. *P* values lower than 0.05 regarded an overall cophylogenetic congruence in the whole dataset. Moreover, a host-parasite link with a squared residual value lower than the global fit was considered as an association with potential cophylogenetic congruence. PACo was implemented in R using the ape [35] and vegan packages [36].

ParaFit, which addresses the fourth-corner problem [30], was performed using the same input data as PACo. Patristic distance matrices were transformed into PCo ordinations and crossed with the host-parasite association matrix. A fourth-corner matrix (D) was generated to compute the ParaFitGlobal statistic. The significance of both the global statistic and individual associations (via ParaFitLink1) was tested with 9,999 permutations and the Cailliez correction for negative eigenvalues, using the Parafit function in the ape R package. Similarly, *p* values lower than 0.05 suggested an overall significant cophylogenetic association between the studied hosts and parasites. Sample size was not calculated since statistical significance is given by posterior probability values of phylogenetic trees, as well as randomizations and jack-knife procedures for PACo analysis.

### Event-based analysis

To further investigate host-parasite coevolution, we used the event-based software eMPRess to estimate the number and type of evolutionary events (e.g., cospeciation, duplication, host switches, and losses) needed to explain the observed associations. This analysis was carried out for the host-parasite groupings with vertebrate and sand fly hosts. Event costs were defined as follows: loss = 1.00, cospeciation = 0.00, and duplication and transfer were determined based on the Costscape plot. These values corresponded to the histogram region with the highest number of most parsimonious reconstructions (MPRs) and the lowest duplication and transfer costs. Input files included.newick trees and a.mapping file of host-parasite associations. The output consisted of visualizations of host and parasite trees with potential coevolutionary scenarios. In addition to eMPRess, Jane was used for computing *Leishmania*-sand fly host coevolutionary events since this dataset included several parasites with multi-host associations. In this case, different *Leishmania* isolates were added to the analysis to avoid false failure-to-diverge estimations. Different host switch event costs (2.00, 3.00, 4.00 and 5.00) were tested in Jane while keeping the loss = 1.00, cospeciation = 0.00, and duplication = 1.00 values constant, with 300 generations and 100 population size. Significance was tested with the costs obtained in 500 solutions.

## Results

### Sequence availability analysis

Since 2010, a total of 26,716 articles related to *Leishmania* have been published in PubMed*.* The *Leishmania* subgenus accounts for 88.12% (n = 23,542) of these publications, followed by *Viannia* with 10.14% (n = 2,709), *Sauroleishmania* with 0.96% (n = 258) and *Mundinia* with 0.77% (n = 207) (S1a Fig and S3 Table). When considering sequence search of specific ribosomal, kDNA and nuclear markers, *L. infantum* had a total of 3,948 sequences deposited in GenBank (S1d Fig), followed by *L. donovani* (n = 2,434)*, L. tropica* (n = 1,922), *L. major* (n = 1,716) and *L. braziliensis* (n = 1,438). Species with less than 10 ribosomal, kDNA or nuclear markers belonged mainly to the *Mundinia* and *Sauroleishmania* subgenus. Among the searched markers, the ITS1, 5.8S and kDNA had the highest number of deposited sequences (n = 3,302, 3,190 and 2,971, respectively) (S4 Table) and varied among *Leishmania* subgenera (S1c Fig). For instance, kDNA has been widely used in studies of the *Viannia* subgenus, while ITS1 has been more commonly applied to *Mundinia.* In contrast, ITS1 and 5.8S loci have been used in roughly equal proportions for investigating the *Leishmania* and *Sauroleishmania* subgenera.

### Phylogenetic trees of 18S, ITS1 and POLR2

**Fig 2:**
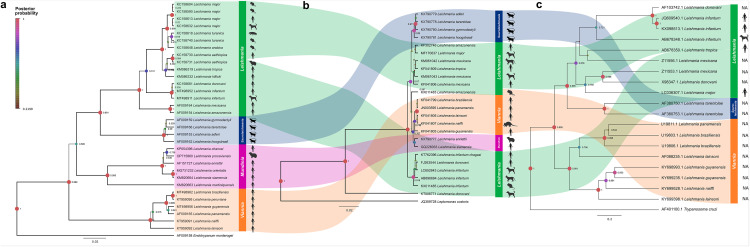
Bayesian inference phylogenetic trees of *Leishmania* spp. using the RNA polymerase subunit II (a), 18S (b) and kDNA (c). Posterior probability (PP) values lower than 0.6 are not shown. Node circle size and color are proportional to the PP. Created in BioRender. Mora, **J.** (2026) https://BioRender.com/is02atg.

Phylogenetic trees shown in Fig 2 were built using 18S, kDNA and POLR2 data, since these markers had the largest number of *Leishmania* spp. represented in GenBank and sequences aligned as a block to the other ones Fig 2). The POLR2 dataset included 26 *Leishmania* spp. of the four subgenera (Fig 2a). Even though sequence OR695081 was available for *L. ellisi,* it could not be included in the analysis since it did not overlap with the same gene fragment. In addition, several sequences of other *Leishmania* spp. (e.g., *L. major, L. infantum* and *L. aethiopica*) were added in the alignment since they were obtained from different vertebrate hosts such as rodents, dogs or mustelids. The POLR2 phylogenetic tree clustered the sequences according to the *Leishmania* subgenera into *Leishmania, Sauroleishmania, Mundinia* and *Viannia*. The latter was basal to the other subgenera, whereas the *Sauroleishmania* was paraphyletic to *Leishmania*.

Eighteen *Leishmania* spp. could be included in the phylogenetic analysis of the 18S since not all sequences overlapped as a block (i.e., *L. garnhami, L. aethiopica, L. martiniquensis* and *L. peruviana*) or were not available in GenBank for others (i.e., *L. ellisi, L. killicki, L. turanica, L. chancei, L. macropodum* or *L. martiniquensis*) (Fig 2b). Furthermore, only *L. enriettii* and *L. siamensis* for the *Mundinia* subgenus and six parasites of the *Leishmania* subgenus were available for the phylogenetic analysis. The *Leishmania* subgenus had a polytomy in the phylogenetic tree with the *Viannia* and *Mundinia* subgenera between them. Additionally, the *Sauroleishmania* subgenus was the most diverged group, whereas *L. infantum* and *L. donovani* were located basally in the tree.

The kDNA dataset included 12 *Leishmania* spp. that met the inclusion criteria for the analysis (Fig 2c). Unfortunately, no sequences of the *Mundinia* subgenus were available in GenBank, five of the *Leishmania* subgenus could align as a block and only one species of the *Sauroleishmania* (i.e., *L. tarentolae*) could be analyzed. The tree topology was similar to the one obtained with the POLR2, with the *Viannia* subgenus basal and *Sauroleishmania* paraphyletic to the *Leishmania* subgenus

The POLR2 dataset was chosen for further cophylogenetic analyses for several reasons. Firstly, it contained the highest numbers of represented *Leishmania* spp. Second, the obtained phylogenetic tree showed a resolution equal to other ribosomal markers and compatible with other studies [11,37]. Third, each subgenus was separated between them with robust posterior probability values and lastly, the available sequences could overlap to the majority.

### Analysis with vertebrate hosts

**Fig 3:**
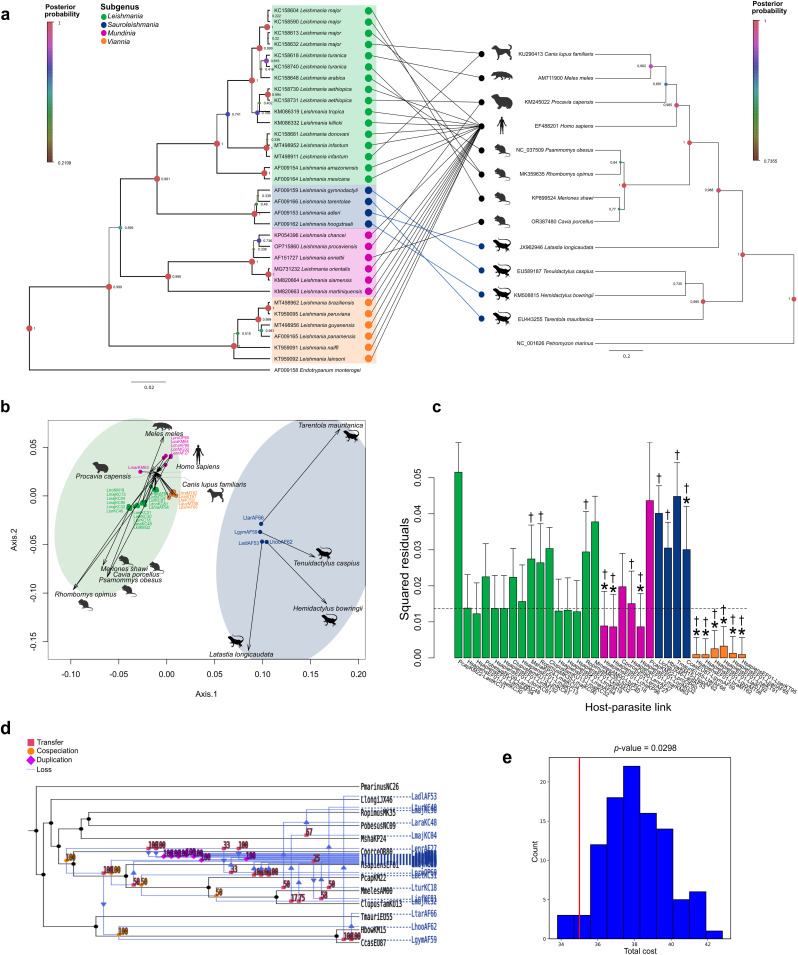
Global-fit and event-based cophylogenetic analysis between *Leishmania* spp. and its annotated vertebrate hosts including *L. gymnodactyli* association. **A.** Tanglegram showing host-parasite associations according to GenBank metadata. **B.** Procrustean superimposition plot between the principal coordinates derived from patristic distances of the RNA Polymerase II of *Leishmania* spp. and their vertebrate host phylogenies. Each parasite and host are denoted as circles and arrow heads, respectively. *Leishmania* spp. are color coded according to the subgenus. Close host and parasite positions in the PCo may indicate cophylogenetic associations **C.** Contribution of each *Leishmania*-vertebrate host link to the global phylogenetic congruence. Each bar represents the squared residual of each association and are color-coded according to the *Leishmania* subgenus. Error bars correspond to 95% confidence intervals of the squared residuals. The median squared residual is indicated as a dotted line. Asterisks at the top of each bar represent a significant ParaFitLink1 value and daggers to significant ParaFitLink2 values. Squared residual values lower than the median squared value suggests cophylogenetic congruence between that host-parasite association. **D.** Coevolutionary reconstruction of the host (black lines) and parasite (blue lines) phylogenies with the lowest global cost according to eMPRess. Values in top of nodes correspond to the support of the predicted event. **E.** Total cost distribution of random solutions. Created in BioRender. Mora, **J.** (2026) https://BioRender.com/is02atg.

Two analyses were conducted to confirm the cophylogenetic relationships between *Leishmania* spp. and their vertebrate hosts. The first analysis included 25 different *Leishmania* spp. and a total of 28 host-parasite links confirmed in GenBank metadata (S2a Fig). The second analysis included 26 *Leishmania* spp. due to the addition of *L. gymnodactyli* associated with the Caspian bent-toed gecko (Fig 3a) and 32 host-parasite links*.*

#### Confirmed *Leishmania*-host parasite links.

*Global-fit analysis:* An overall significant phylogenetic congruence was obtained between the confirmed *Leishmania-*vertebrate host links with a *p* value of 0.0001 and a m^2^_XY_=0.5109, summarized in Table 1. The superposed PCA of host and parasite genetic distances showed the *Sauroleishmania* spp. (i.e., *L. adleri, L. hoogstraali* and *L. tarentolae*) separated from the other species and closer to the reptile hosts *Latastia longicaudata, Hemidactylus bowringii* and *Tarentola mauritanica,* indicating a closer cophylogenetic relationship. On the other hand, species of the *Leishmania, Viannia* and *Mundinia* subgenera were located in a separate cluster with their respective mammal hosts (S2b Fig). Five host-parasite links were below the median squared residual value, specifically the associations between *Homo sapiens* and *L. braziliensis, L. guyanensis, L. lainsoni, L. panamensis* and *L. peruviana.* The highest squared residual values corresponded to the association between *L. turanica* and the great gerbil *Rhombomys opimus* and *L. hoogstraali* and the oriental leaf-toed gecko *Hemidactylus bowringii* (S2c Fig).

**Table 1:** Summary of statistics of the global fit and event-based methods for the analysis of coevolution between *Leishmania* spp. and its vertebrate and invertebrate hosts.

Analysis	Best nucleotide substitution models (parasite tree, host tree)	PACo (m^2^_XY_ value, p value)	ParaFit (*p* value, ParaFit Global, number of significant links)	Event-based results (*p* value, number of events)
*Leishmania* and its vertebrate hosts, strict*	TN93 + G, GTR + G	0.5109, 0.0001	*p* = 0.005, 0.009, 12	*p* = 0.0269, 3 cospeciations, 17 duplications, 10 host-switches and 0 losses
*Leishmania* and its vertebrate hosts, with L. gymnodactyli**	TN93 + G, GTR + G	0.5419, 0.0001	*p* = 0.0034, 0.012, 11	*p* = 0.0269, 5 cospeciations, 7 duplications, 19 host-switches and 0 loss
*Leishmania* and its invertebrate hosts***	TrN + G, TIM2 + G	1.3988, 0.0001	*p* = 0.0001, 0.062, 39	*p* = 0.049, 8 cospeciations, 2 duplications, 11 host-switches and 15 losses^1^ *p* = 0.01, 19 cospeciations, 9 duplications, 24 host-switches and 38 losses^2^

*Took into consideration only those associations annotated in GenBank

**All associations were derived from GenBank, except the one between *L. gymnodactyli* and *Tenuidactyus caspius*, which according to phylogenetic closeness to other reported hosts, may be possible.

***Possible associations according to the literature.

^1^Event-based analysis of *Leishmania* and its invertebrate hosts computed in eMPRess.

^2^Event-based analysis of different *Leishmania* sequences and their invertebrate hosts computed in Jane.

The Parafit function revealed a significant cophylogenetic relationship, with a *p* value of 0.005 and a ParaFitGlobal statistic of 0.009. In addition, twelve host-parasite links were considered to contribute to the significant pattern of coevolution as obtained in ParaFitLink1 (F1) *p* values (Additional Fig 1c). These associations concerned all those with the *Viannia* subgenus (n = 6/6), four out of six links of the *Mundinia* subgenus, one from the *Leishmania* subgenus (*L. major* with *Rhombomys opimus)* and one of the *Sauroleishmania* subgenus (*L. hoogstraali* with *H. bowringii*).

*Event-based analysis:* The event-based analysis using eMPREss found a significant coevolutionary association between *Leishmania* parasites and its vertebrate hosts mainly driven by duplication (i.e., speciation of the parasite without host divergence; Additional Fig 1d and 1e, *p* = 0.0396). Seventeen duplication events were predicted with high confidence, all of them concerning *Leishmania* spp. parasitizing humans, like *L. amazonensis, L. donovani, L. mexicana, L. killicki,* and species of the *Mundinia* (with the exception of *L. procaviensis*) and *Viannia* subgenus (S2d Fig). Host switching or transfer was calculated in 10 situations. Some examples were the potential switches of *L. tarentolae* from *T. mauritanica* to generate *L. adleri* in the common long-tailed lizard *L. longicaudata*, the transfer of *L. turanica* from *R. opimus* to the European badger *Meles meles*, the switch of *L. major* from *R. opimus* to humans, *L. procaviensis* from humans to the hyrax *Procavia capensis*, or *L. infantum* from humans to the domestic dog *Canis lupus familiaris.* In addition, three cospeciation events were observed, namely in the divergence of *L. tarentolae* with the Moorish gecko *Tarentola mauritanica* and *L. hoogstraali* and *H. bowringii*, and two events involving the separation of *L. major* from *L. turanica* and *L. arabica* with their rodent hosts. Finally, no losses were observed, meaning that hosts did not diverge without concomitant parasite speciation.

#### *Leishmania*-host parasite links with *L. gymnodactyli.*

*Global-fit analysis:* The second analysis of *Leishmania* spp. with its vertebrate hosts including *L. gymnodactyli* (Fig 3a) improved the resolution of the analysis and confirmed the results obtained in the strict vertebrate host database S2 Fig. An overall significant cophylogenetic correspondence was obtained in PACo (*p = *0.0001, m^2^_XY_=0.5419) and ParaFit (*p = *0.0034, ParaFit Global statistic = 0.012) (Table 1). As in Additional Fig 1, two clusters were observed in the PCoA (Fig 3b): one with *Sauroleishmania* spp. and its reptile hosts and a second group with *Leishmania, Viannia* and *Mundinia* spp. with its mammal hosts. The residual cost plot showed the same host-parasite links with a value lower than the median square residual value (Fig 3c), like *Viannia* and *Mundinia* spp. with their hosts, except for *L. martiniquensis* with humans and *L. procaviensis* with *P. capensis*, and *L. gymnodactyli* with *T. caspius.* Furthermore, the host-parasite links with the highest squared residuals values corresponded to *L. aethiopica* and *L. procaviensis* associated with the hyrax *Procavia capensis.* Even though *L. infantum* and *L. tropica* have also been associated with *P. capensis*, POLR2 sequences obtained from infected hyraxes could not be obtained.

**Fig 4:**
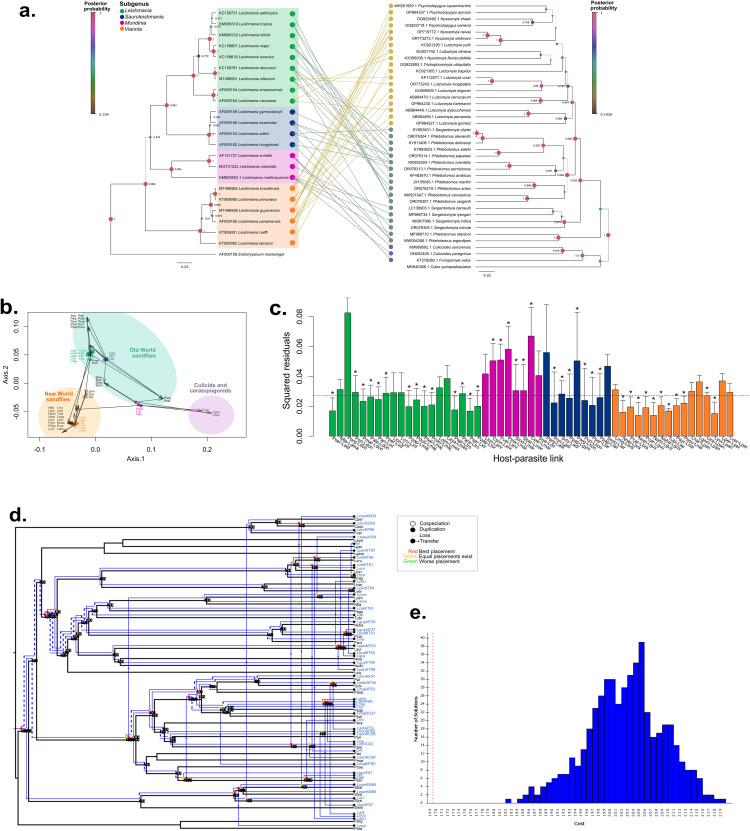
Global-fit and event-based cophylogenetic analysis between *Leishmania* spp. and its invertebrate hosts. **A.** Tanglegram showing possible host-parasite associations according to the literature. **B.** Procrustean superimposition plot between the principal coordinates derived from patristic distances of the RNA Polymerase II of *Leishmania* spp. and their invertebrate host phylogenies. Each parasite and host are denoted as circles and arrow heads, respectively. *Leishmania* spp. are color coded according to the subgenus. Close host and parasite positions in the PCo may indicate cophylogenetic associations **C.** Contribution of each *Leishmania*-invertebrate host link to the global phylogenetic congruence. Each bar represents the squared residual of each association and are color-coded according to the *Leishmania* subgenus. Error bars correspond to 95% confidence intervals of the squared residuals. The median squared residual is indicated as a dotted line. Asterisks at the top of each bar represent a significant ParaFitLink1 value and daggers to significant ParaFitLink2 values. Squared residual values lower than the median squared value suggests cophylogenetic congruence between that host-parasite association. **D.** Coevolutionary reconstruction of the host (black lines) and parasite (blue lines) phylogenies with the lowest global cost obtained with Jane taking into consideration different *Leishmania* spp. isolates when having multi-host parasites. Values on top of nodes correspond to the support of the predicted event **E.** Total cost distribution of random solutions obtained with Jane. Red dotted line indicated the cost of the analyzed solution. Animal icons were created with Biorender.com.

The ParaFit function demonstrated a strong cophylogenetic relationship with 11 significant links according to the ParaFitLink1 (F1) *p* value and 18 with the ParaFitLink2 (F2) *p* values (Fig 4c). The significant links according to ParaFitLink1 corresponded to the same host-parasite links obtained for the strict vertebrate host database (S2 Fig). In addition to these, the ParaFitLink2 determined as significant host-parasite associations from the *Viannia, Mundinia* and *Sauroleishmania* and three links of the *Leishmania* subgenus, i.e., *L. major* with *Meriones shawi* and *R. opimus* and *L. turanicus* with *R. opimus* (Fig 3c).

*Event-based analysis:* The coevolutionary solution provided in the analysis between *Leishmania* spp. with its vertebrate hosts, including *L. gymnodactyli*, suggested that duplication and host-switching were the most frequent events (Fig 3d). Moreover, the solution provided was significant by taking into consideration the costs obtained from 500 random samples (*p* = 0.0298) (Fig 3e). Duplications were predicted in seven host-parasite associations involving *Leishmania* parasites with humans. In addition, host switches were suggested in *L. aethiopica* from hyraxes to humans, *L. infantum* from humans to dogs or *L. turanica* from the European badger to rodents*.* Interestingly, a host switch was predicted, with *L. gymnodactyli* (associated with *T. caspius*) transitioning to become *L. tarentolae* (associated with *T. mauritanica*). Indeed, a cospeciation event resulted in the divergence between *L. gymnodactyli* from *L. tarentolae* and *L. hoogstraali* with their reptile hosts. In addition, a cospeciation event was predicted at a higher taxonomical level in the split of mammals from lizards with the consequent separation of *Leishmania, Viannia* and *Mundinia* subgenus from *Sauroleishmania.* Furthermore, another cospeciation was predicted in the divergence of *L. turanica, L. aethiopica, L. major* and *L. infantum* with domestic dogs, the European badger and hyraxes of the other *Viannia, Leishmania* and *Mundinia* spp. cospeciating with rodents and humans. One loss was estimated in *L. major* with the speciation of the rodents *R. opimus* from *M. shawi,* without leading to an additional *Leishmania* spp.

### Analysis with invertebrate hosts

The study of the association between *Leishmania* and its invertebrate hosts (i.e., sand flies, biting midges) resulted in a strong cophylogenetic congruence. The tanglegram shows the association of *Leishmania* spp. distributed in the Americas, like species of the *Viannia* subgenus, *L. infantum, L. amazonesis* and *L. mexicana,* to the New World sand fly genera *Lutzomyia, Nyssomya, Trichophoromya* and *Psychodopygus* (Fig 4a). Conversely, biting midges were linked to the *Mundinia* subgenus, whereas Old World sand flies of the *Phlebotomus* and *Sergentomyia* subgenera to *Sauroleishmania* and *Leishmania* (Fig 4a).

#### Global-fit analysis.

As with vertebrate hosts, the PACo (m^2^_XY_=1.3988, *p* = 0.0001) and ParaFit functions (ParaFit Global = 0.062, *p = *0.0001) showed a cophylogenetic congruence between *Leishmania* and invertebrate hosts (Table 1). The PCoA superimposing host and parasite genetic distances in Fig 4b mirrored the tanglegram observations and grouped host-parasite links into three clusters: i) species of the *Viannia* subgenus, together with *L. mexicana, L. amazonensis* and *L. infantum* associated to New World sand flies, ii) *Mundinia* parasites associated to mosquitoes and biting midges and iii) species of the *Leishmania*, *Mundinia* and *Sauroleishmania* subgenera associated to Old World sand flies. The squared residual analysis revealed eight host-parasite links below the mean squared residual value (Fig 4c). Accordingly, the association between *L. aethiopica* and *L. killicki* with *Phlebotomus sergenti*, *L. braziliensis* and *L. guyanensis* with *Nyssomyia neivai*, *L. guyanensis* with *Nyssomyia whitmani*, *L. lainsoni* with *Trichophoromyia ubiquitalis*, *Leishmania naiffi* with *Psychodopygus squamiventris*, and *L. panamensis* with *Lutzomyia yuilli*. Conversely, the highest squared residual values were obtained with *L. donovani* and *L. adleri* with *Phlebotomus argentipes*.

The Parafit function found 39 significant links according to the ParaFitF1 statistic: 70% (14/20) corresponding to the *Leishmania* subgenus, 75% (6/8) to the *Mundinia*, 78% (7/9) in *Sauroleishmania*, and 69% (11/16) of *Viannia*. Non-significant host-parasite corresponded to those with *L. mexicana* and New World sand flies *Lutzomyia olmeca* and *Lutzomyia ayacuchensis*, as well as *L. amazonensis* with *Nyssomyia flaviscutellata, L. infantum* with *Lu. longipalpis* and *Lutzomyia cruzi, L. enriettii* with *Lu. longipalpis*, *L. adleri* with *P. argentipes*, *L. tarentolae* with *Sergentomyia minuta, L. braziliensis* with *Lutzomyia migonei*, as well as *L. panamensis* with the sand flies *Lutzomyia gomezi*, *Lutzomyia hartmanni,* and *Lutzomyia peruensis*.

#### Event-based analysis.

Coevolutionary events were predicted first with eMPRess, which estimated 8 cospeciations, 2 duplications, 11 transfers and 15 losses (S3a Fig). These results were statistically significant (*p* = 0.049), meaning that the total cost of the obtained solution did not fall into other randomly calculated scenarios (S3b Fig). Interestingly, this solution failed to include those associations for multi-host *Leishmania* spp., like *L. infantum* linked with *Phlebotomus ariasi, Phlebotomus perniciosus, Lu. longipalpis* and *Lutzomyia cruzi*. For this reason, Jane algorithm was employed to accurately analyze multi-host parasites, and estimated 19 cospeciations, 9 duplications, 24 transfers and 38 losses (Fig 4d).

Cospeciation events were predicted at higher taxonomical levels in the divergence of New World sand flies from the Old World sand flies with *Viannia* subgenus species and *Leishmania, Sauroleishmania* and *Mundinia* subgenus species, respectively, or the divergence of *Sergentomyia* sand flies with *L. tarentolae, L. martiniquensis* and *L. orientalis* with the other sand fly-*Leishmania* links. Moreover, duplications were observed in those *Leishmania* spp. from the same sand flies such as *L. guyanensis* and *L. braziliensis* in *Psychodopygus carrerai,* or *L. gymnodactyli* and *L. tarentolae* in *P. papatasi.* In addition, host-switching was predicted for multi-host parasite species, including *L. infantum* (in *P. perniciosus, Lu. longipalpis* and *P. ariasi*), *L. panamensis* (from *Lu. yuilli* to *Lu. gomezi, Lu. harmanni* and *Lu. trapidoi*)*,* or *L. tarentolae* (from *P. papatasi* to *P. perniciosus* and *Sergentomyia minuta*). Finally, parasite losses were estimated at higher taxonomical levels where branching of sand flies was observed without divergence of *Leishmania*. This solution was significant since the cost was lower than the randomly obtained solutions (Fig 4e).

## Discussion

Data suggest that *Leishmania* spp. coevolved with their vertebrate and invertebrate hosts, being mostly characterized by events of parasite-sand fly cospeciation, as well as host switching and duplication in their associations with vertebrate hosts. Overall, findings support the Supercontinent hypothesis enhancing current understanding of *Leishmania* origins and diversification, with an emphasis to the adaptation occurred amongst vertebrate hosts and vectors, under the frame of a complex digenetic life cycle.

The most abundant and represented molecular markers for *Leishmania* (i.e., POLR2, 18S and kDNA loci) shared the same characteristics, having a general congruence (i.e., similar rate of evolution between them) in the inferred phylogenetic trees built with markers of different cellular origin (i.e., nuclear, mitochondrial and ribosomal). Choosing the right marker is essential during phylogenetic reconstructions since markers with low intra-specific variation, such as HSP70, may yield low resolution tree branching and lead to improper conclusions during cophylogenetic analyses. On the other hand, highly polymorphic loci like kDNA provide more information regarding species divergence [38]. In this context, mutation rates of POLR2 have not been reported; however, the species branching patterns in the phylogenetic trees shown in Fig 2 were comparable to those obtained with kDNA, demonstrating a similar phylogenetic resolution for both markers.

Phylogeny based on POLR2 was similar with trees constructed before [21,39,40], also, this dataset included 26 species, compared to the 18S gene, which comprised 18 species and only 12 species in the kDNA. In this study, as well as in those above referred, *Leishmania* and *Sauroleishmania* were identified as the most divergent groups*.* However, *Viannia* was basal in our phylogenetic tree, as opposed to other reports [40], where *Mundinia* has been basal to the other subgenera, probably due to different sequence lengths, type of dataset used and methodological approach, in each analysis. For instance, POLR2 sequences used by Kwakye-Nuako, Mosore (40) were 1,200 long, the tree built by Sapp, Low (39) concatenated other nuclear and mitochondrial markers, whereas the one by Harkins, Schwartz (21) contained 200,000 informative sites across *Leishmania* genomes. Nevertheless, cophylogenetic analyses consider phenetic or patristic distances between matched host and parasite taxa [29,30], therefore, the differential positioning of a cluster should not bias the results.

An overall significant phylogenetic congruence was obtained between *Leishmania* spp. and their vertebrate hosts, which might have derived from the cospeciation between the *Sauroleishmania* subgenus and lizards, and mammals with the *Viannia*, *Leishmania* and *Mundinia* subgenera. In particular, cophylogenetic analysis suggests a strong relationship between species of the *Viannia* subgenus and humans, since all host-parasite links were significant. Accordingly, humans should be the origin source of this subgenus in the New World and of the *Leishmania* subgenus in the Old World, representing the main reservoir of the parasites in domestic cycles [41]. Nonetheless, a comprehensive cophylogenetic analysis is impaired by the absence of POLR2 sequences of *Viannia* species in two-toed sloths (*Choloepus hoffmanni*), domestic dogs as well as opossums, all species found positive for this subgenus [42]. This cophylogenetic signal would have provided valuable insights into the role of sloths as ancient hosts of *Leishmania*, as the Neotropical hypothesis of *Leishmania* origin suggests that these animals served as the primary reservoirs of the parasites [43]. Therefore, the current findings highlight the phylogenetic congruence of *Viannia* parasites and humans, but their role as main reservoirs of *Leishmania* (*Viannia*) needs further investigations. Hence, reservoir competence and infectiousness studies in different animal hosts deserve to be explored [44].

In addition, data herein obtained discard the Palearctic hypothesis, which postulates that *Sauroleishmania* is basal to [22] and an ancestor of other *Leishmania* spp., according to ecological, biochemical and biogeographical evidence [45]. The above is explained by two reasons: i) POLR2 phylogeny places the *Sauroleishmania* cluster as a sister group to *Leishmania* and *Mundinia,* as observed in other studies [21,39,40]*,* while our data place *Viannia* basal to the other groups; ii) our results show the cospeciation of lizards with *L. adleri, L. tarentolae, L. gymnodactyli* and *L. hoogstraali*, probably supported by long-term associations. Therefore, if *Sauroleishmania* emerged from mammals, as the Palearctic hypothesis states, the associations with herpetofauna would be recent and thus, parasites less adapted to these hosts. Nonetheless, this study and epidemiological evidence shows *Sauroleishmania* infecting primarily reptiles [46]. In addition, infection by this group is transient [47] and asymptomatic in rodents [48], dogs [14] and humans [49], suggesting the low adaptation of *Sauroleishmania* to mammal hosts.

**Fig 5:**
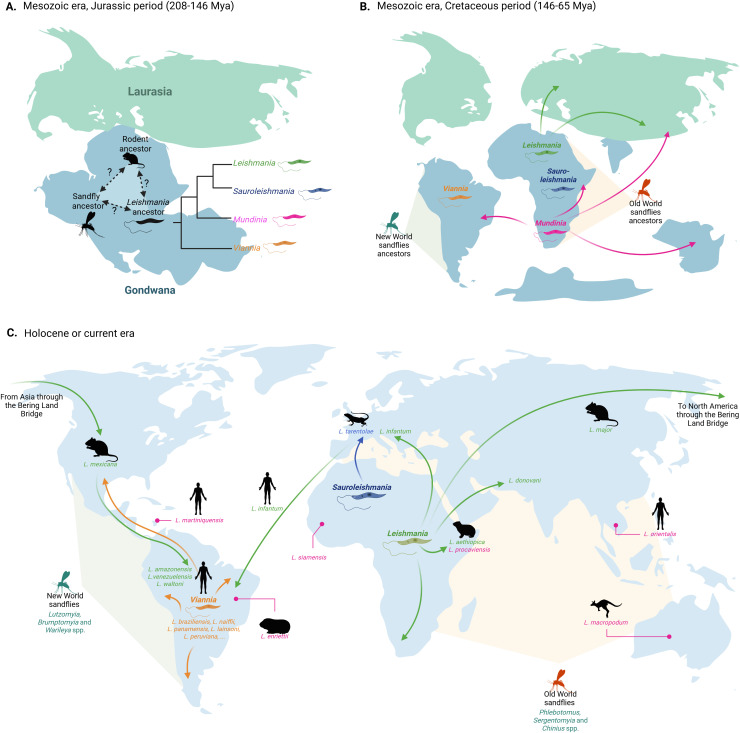
Supercontinent or Multiple origin hypothesis on the origin of the genus *Leishmania* according to the results obtained in this research, and previous observations made by Steverding (53), Harkins, Schwartz (21), Akhoundi, Kuhls (12) and Momen and Cupolillo (24). **A.** During the Mesozoic era in the Jurassic period, before the breakup of the supercontinent Gondwana, *Leishmania* parasites may have used ancient rodent species to co-diversify, while ancient sand fly species may have emerged earlier. **B.** After the split of Gondwana in the Cretaceous period, different *Leishmania* subgenera arose, i.e., *Viannia* in South America, *Leishmania* and *Sauroleishmania* in Africa and *Mundinia* spread all over the world. With time, speciation in vertebrate hosts occurred by host-switching or duplication, where ancestral parasite species adapted to permissive hosts giving rise to new *Leishmania* spp. and parasites cospeciated with ancestors of the New and Old World sand flies. **C.** Dispersal of *Leishmania* spp. by using different vertebrate and invertebrate host gave rise to the colonization of new geographical regions. For instance, it has been suggested that rodents infected with *L. major* migrated through the Nearctic by the Bering Land Bridge to North America, originating *L. mexicana* in autochtonous rodent species, which further migrated to the South giving rise to *L. amazonensis, L. venezuelensis* or *L. waltoni*. Moreover, *L. aethiopica* was established in East Africa with hyraxes, as well as ***L.*** *(Mundinia) procaviensis*, and then switched to humans. Species of the *Viannia* subgenus spread throughout South and Central America using humans as a main reservoir, but according to other studies, sloths and porcupines may have played an important role in the maintenance of ancestral *Viannia* spp. In addition, species of the *Sauroleishmania* subgenus spread with herpetofauna to North Africa, East Asia and Europe, without having been reported in the Americas. Finally, species of the *Mundinia* subgenus have been described in diverse and widely separated geographical regions, suggesting that their distribution may date back to before the breakup of Gondwana. Created in BioRender. Mora, **J.** (2026) https://BioRender.com/jzxajbr.

A significant cophylogenetic signal (e.g., between *L. major* with *M. shawi* and *R. opimus* and between *L. turanicus* with *R. opimus*) was obtained between parasites of the *Leishmania* subgenus and rodent hosts. Accordingly, many rodents (subfamily: Arvicolinae, Callosciurinae, Cricitinae, Gerbillinae, Murinae and Sciurinae) may be infected by species of the *Leishmania* subgenus, with prevalence of up to 39.1% [50]. This may suggest that *Leishmania* subgenus is more established in rodents, as these were one of the first placental mammals developing during the Paleocene (~61.7-62.4 Mya), after the split of the supercontinent Gondwana in the late Mesozoic era [12,51,52]. Indeed, the Supercontinent hypothesis supports that *Leishmania* started infecting rodents during the Mesozoic era on the supercontinent Gondwana [53] (Fig 5). Thus, data herein reported and illustrated in Fig 5 supports this observation since long-term host-parasite associations usually have better co-adaptations, as observed in other protozoa infections [54]. Conversely, non-significant cophylogenetic relationships were obtained between species of the *Leishmania* subgenus and other mammalian hosts (i.e., human, mustelid, canid, felid or hyrax), which suggests more recent host-parasite relationships [12]. For instance, mustelids originated around 33 Mya [55], canids 40 Mya [56], and felids around 15–10 Mya [57], possibly indicating that the subgenus *Leishmania* initially infected rodents and later switched or duplicated to these other hosts, as confirmed in our study. After the split of Gondwana, approximately 180–50 Mya, species of the genus *Leishmania, Sauroleishmania* and *Viannia* suffered from geographical isolation, and therefore, started to quickly diversify over the years [53] (Fig 5).

The Supercontinent hypothesis is also supported by the fact that hyraxes serve as reservoirs for *L. aethiopica* in Ethiopia and Kenya, reinforcing the idea of an African origin for the *Leishmania* subgenus [24]. Indeed, a cospeciation event was predicted in our study between the hyrax *P. capensis* and *L. aethiopica* as well as *L. procaviensis*. The origin of hyraxes is estimated to have occurred between 30–25 Mya [58], which aligns with the divergence of *L. aethiopica* around 10 Mya [21] and the origin of *L. procaviensis*. Hyraxes have also been found with *L. tropica* in other countries, such as in Israel [59], which might reveal a potential host-switching from humans to these hosts. A hypothesis that needs to be confirmed as target sequences from *L. tropica* infecting hyraxes were not available for the study. The role of hyraxes as natural reservoirs of the parasite was also inferred by the host switching events of *L. aethiopica* from hyraxes to humans, though a clear cophylogenetic signal between hyraxes and *L.* (*Leishmania*) *aethiopica* and *L.* (*Mundinia*) *procaviensis* was not obtained. This may be explained by the different phylogenetic positions of these two *Leishmania* spp. to distinct subgenera, or to the relatively recent divergence of *L. aethiopica* [21], resulting in a short host-parasite coevolutionary history. These findings highlight the temporal alignment between hyrax evolution and the origin of *L. aethiopica* and support a broader pattern of host–parasite co-divergence.

The present study estimated that cospeciation was the primary driver for coevolution between *Leishmania* and sand flies with 19 calculated events, a strong cophylogenetic signal with most host-parasite links and a clear clustering of Old World sand flies with *Leishmania, Sauroleishmania* or *Mundinia* parasites, and New World sand flies with the *Viannia* subgenus (Fig 4b). More than 800 sand fly species have been described in the world, 464 Neotropical species [60] and 375 recognized in the Old World [61]. Sand flies exhibit clear biogeographical differences, with the genera *Phlebotomus, Sergentomyia* and *Chinius* found in the Palearctic, Africa, the Mediterranean, Asia and Australia, whereas the *Lutzomyia, Brumptomyia* and *Warileya* genera are mainly restricted to the Americas [12]. Of these, only a few sand fly species are permissive to *Leishmania* development, as an effect of the adaptation to specific metabolic or physiological characteristics of these arthropods, such as salivary protein families [62], or protein ligands in the sand fly midgut [63,64]. This geographical segregation and intrinsic biological differences between sand flies, may contribute to the significant cophylogenetic relationship.

According to our findings, phylogenies between *Leishmania* spp. and their sand fly hosts were globally congruent. The separation of New and Old World sand flies has been estimated to have occurred after the breakup of Gondwana, ~ 200 Mya, (Fig 5). However, other studies have suggested that the split of these two groups occurred before, during the Triassic period when Pangea was divided into Laurasia and Gondwana giving rise to Old and New World sand flies, respectively [65]. If this was the case, parasites of the *Leishmania, Sauroleishmania* or *Mundinia* could have used New World sand fly species as hosts and coadapted to them during the Mesozoic era. However, we found no cophylogenetic congruence between *L. infantum*, *L. mexicana* and *L. amazonensis* and New World sand flies according to the individual squared residual values. On the other hand, host-switching of *L. infantum* was estimated from *Lutzomyia* spp. (*Lu. longipalpis* and *Lu. gomezi*) to *Phlebotomus* spp. (*P. perniciosus* and *P. ariasi*), suggesting the adaptation of *Leishmania* subgenus first to New World sand flies. As a further support to the latter observation, the New World sand fly *Lu. longipalpis* has been demonstrated as a competent host of the Old World *L. major,* possibly facilitated by the involvement of unspecific host-parasite interactions [66]. To unravel the origin of sand flies and their potential association with *Leishmania* spp., further time-calibrated phylogenetic trees using additional loci or genomic data should be rigorously analyzed.

*Leishmania enrietti* and *L. martiniquensis*, belonging to the *Mundinia* subgenus, were found with phylogenetic congruence with biting midges such as *Culicoides sonorensis* and *Culicoides peregrinus,* as well as *Forcipomyia velox*. In addition, *L. (Mundinia) macropodum* has been reported in the literature in the biting midge *Forcipomyia* sp. [67] and *L. orientalis* in *C. sonorensis* [68]*.* The evolutionary history of *Mundinia* remains largely unknown due to its recent classification following its description [15]. Nevertheless, parasites may be adapted to New and Old World sand flies, as well as biting midges, because of the ancient global distribution of *Mundinia* since before the breakup of Gondwana [53] with the subsequent adaptation to various arthropod species (Fig 5). This may have led to the non-specific attachment of the parasites to arthropod-associated molecules in the gut, favoring some vectors over others, such as the case of *L. martiniquensis, L. orientalis* and *L. siamensis,* which successfully colonize *C. sonorensis*, rather than *Phlebotomus argentipes* [69]. Importantly, *Mundinia* subgenus is still unexplored in terms of the pathogenic mechanisms it induces in hosts, host-parasite interactions and its phylogeographic distribution, all of which are crucial for gaining deeper insights into the origin of *Leishmania*.

Overall, coevolution between *Leishmania* and vertebrate hosts was estimated to be mainly driven by host-switching and duplication, whereas cospeciation appeared to be the most frequent event occurring in the *Leishmania-*invertebrate host. Host-switching occurs when a parasite adapts to a new host species, gradually reducing its reproductive interaction with the original host [70]. This process may result from ecological fitting, where pre-existing traits allow parasites to infect new permissive hosts, leading to novel associations [71]. If the new relationship proves successful and genetic exchange with the ancestral population decreases, speciation can occur, a process that often spans hundreds of thousands of years [72]. The divergence between *Paraleishmania* and *Euleishmania* spp. was estimated at approximately 90 Mya [21], and the association with ancient rodents and sand flies dates back to the Mesozoic era, between 280–100 Mya (Fig 5) [53], a time span sufficient to account for the current diversification of *Leishmania* spp. On the other hand, speciation by duplication occurs without a host change, as parasites diverge within the same host due to mutational changes that, over time, lead to the formation of new taxa [73]. In this sense, some invertebrate and vertebrate hosts have been reported with various *Leishmania* spp. [4]. For instance, humans are proven efficient hosts, producing overall mild clinical manifestations that, depending on the *Leishmania* spp., usually resolve with treatment [8,74], and other animal hosts may be infected without associated clinical signs [75]. The mild clinical presentation associated to leishmaniasis may stem from the evolutionary and immunological balance between parasites and hosts, where limited damage benefits the parasite’s effective transmission, as demonstrated with *L. tarentolae* [76–78]. In addition, *L. infantum* has been associated with post-kala azar dermal signs in human patients from the Old World [79], but these manifestations have never been reported in the Americas. This suggests that additional players, such as genetic hybridization and recombination between populations [80], are taking part in host-parasite interactions together with molecular adaptations and coevolution.

Cophylogenetic analyses are subject to inherent limitations, particularly concerning the availability of GenBank sequences of *Leishmania* spp. Moreover, only host–parasite associations with clearly annotated host origins could be incorporated, thereby constraining the number of taxa included in the analysis. Ideally, all taxa should be included in global-fit and event-based analysis, as well as comprehensive genomic datasets from both hosts and parasites should be employed to achieve more robust evolutionary inferences, as genes may diverge at different rates [29,81]. Nonetheless, such an approach would further restrict the number of host–parasite associations available for analysis, given that numerous species have been not yet sequenced to date. In addition, having the geographical source for each *Leishmania* spp. clearly specified would have provided us relevant information regarding the parasite distribution. Finally, even though host and parasite phylogenies were reconstructed with markers having different evolutionary rates, global-fit methods rely on patristic distances between taxa. In both phylogenies, high branch resolution was observed, indicating that gene origin might have not influenced the results, as described in a previous study with *Hepatozoon* spp. Even though these limitations exist, the conclusions obtained in this study are supported by robust statistical data. Cospeciation was regarded for a long time as the sole mechanism for host-parasite coevolution and supposes the orchestrated diversification of host and parasite phylogenies [70]. The high number of cospeciation events estimated in *Leishmania*-sand fly phylogenies, as opposed to those with vertebrates, suggests that a combination of intrinsic and environmental factors influences parasite attachment, multiplication or transfer to the next host [4]. Moreover, host encounter rates and availability, as well as topographical and climatic conditions may have affected sand flies and ancient mammals differently [22]. In addition, another challenging aspect that may hindrance our understanding of the evolution and divergence of *Leishmania*, is the possibility of facultative sexual reproduction, rather than the well-known cloning [82]. This cryptic, sexual reproduction, apart from complicating the evolutionary models needed to infer the relationship between *Leishmania* an its vertebrate hosts and its invertebrate vector, may lead to the generation of genotypes with unpredictable phenotypes in tissue tropism, virulence, or the emergence of drug resistance [83].

Gaining knowledge of the relationships between *Leishmania* and specific host orders is crucial for effective disease monitoring and control. Screening parasites in sympatric and phylogenetically related species will facilitate the identification of new hosts that may serve as reservoirs or vectors of *Leishmania*, thereby strengthening epidemiological surveillance and reducing the risk of parasite spread into new geographical areas or populations. Altogether, this study demonstrates that, despite millions of years of evolutionary divergence, *Leishmania* remains a highly adaptable parasite, capable of overcoming both biological and environmental challenges to maintain its digenetic life cycle. This comprehensive cophylogenetic analysis enhances our understanding of *Leishmania* origins and diversification, offering valuable insight into host specificity, vector adaptation, and the long-term maintenance of its digenetic life cycle, leading to the development of targeted interventions to mitigate the impact of *Leishmania* infections in at-risk populations.

## Supporting information

S1 Table List of sand fly species that have been reported as suitable hosts for *Leishmania* spp. with the codes used for subsequent analyses. (XLSX)

S2 Table List of *Leishmania* spp. with their strain codes and GenBank accession numbers that have been isolated from various vertebrate species. These species are accompanied by the codes used for subsequent analyses.
(XLSX)

S3 Table Number of scientific publications indexed in Pubmed according to *Leishmania* spp. from 2010 to 2024. (XLSX)

S4 Table Number of genomes and sequences of various nuclear, mitochondrial and ribosomal markers associated with different *Leishmania* spp. (XLSX)

S1 Fig Analysis of the *Leishmania* spp. sequence availability on GenBank to determine the most suitable markers to conduct the analysis. **A.** Number of publications using *Leishmania* keywords according to year and subgenus. **B.** Number of genomes available (light blue bars), total number of sequences (blue lines) and total number of publications (orange lines) according to *Leishmania* spp. **C.** Number of deposited sequences according to molecular marker and *Leishmania* subgenus. **D.** Number of deposited sequences according to molecular marker and *Leishmania* spp.
(TIF)

S2 Fig Global-fit and event-based cophylogenetic analysis between *Leishmania* spp. and its annotated vertebrate hosts. **A.** Tanglegram showing host-parasite associations according to GenBank metadata. **B.** Procrustean superimposition plot between the principal coordinates derived from patristic distances of the RNA Polymerase II of *Leishmania* spp. and their vertebrate host phylogenies. Each parasite and host are denoted as circles and arrow heads, respectively. *Leishmania* spp. are color coded according to the subgenus. Close host and parasite positions in the PCo may indicate cophylogenetic associations. **C.** Contribution of each *Leishmania*-vertebrate host link to the global phylogenetic congruence. Each bar represents the squared residual of each association and are color-coded according to the *Leishmania* subgenus. Error bars correspond to 95% confidence intervals of the squared residuals. The median squared residual is indicated as a dotted line. Asterisks at the top of each bar represent a significant ParaFitLink1 value and daggers to significant ParaFitLink2 values. Squared residual values lower than the median squared value suggests cophylogenetic congruence between that host-parasite association. **D.** Coevolutionary reconstruction of the host (black lines) and parasite (blue lines) phylogenies with the lowest global cost according to eMPRess. Values in top of nodes correspond to the support of the predicted event. **E.** Total cost distribution of random solutions. Created in BioRender. Mora, J. (2026) https://BioRender.com/is02atg.
(TIF)

S3 Fig Event-based analysis of *Leishmania* spp. and its possible invertebrate hosts according to the literature. **A.** Coevolutionary reconstruction of the host (black lines) and parasite (blue lines) phylogenies with the lowest global cost obtained with eMPRess. Values in top of nodes correspond to the support of the predicted event. **B**. Total cost distribution of random solutions obtained with eMPRess. Red dotted line indicated the cost of the analyzed solution.
(TIF)
